# Lipidomic markers of sperm cryotolerance in cattle

**DOI:** 10.1038/s41598-020-77089-9

**Published:** 2020-11-19

**Authors:** Holly C. Evans, Thu T. N. Dinh, Muhammet Rasit Ugur, Mustafa Hitit, Dishnu Sajeev, Abdullah Kaya, Einko Topper, Molly C. Nicodemus, Gary D. Smith, Erdogan Memili

**Affiliations:** 1 Department of Animal and Dairy Sciences, Mississippi State University, Starkville, MS USA; 2 Department of Animal Genetics, Kastamonu University, Kastamonu, Turkey; 3 Department of Reproduction and Artificial Insemination, Selcuk University, Konya, Turkey; 4 URUS Group LP, Madison, WI USA; 5 Departments of Obstetrics and Gynecology, Physiology, Urology, and Reproductive Sciences Program, University of Michigan, Ann Arbor, MI USA

**Keywords:** Cell biology, Developmental biology

## Abstract

The objective of the current study was to determine the fatty acid composition of sperm from Holstein bulls with different freezability (Good and Poor; *n* = 12). Fatty acids were extracted from frozen sperm in 1:2 (v/v) chloroform–methanol solvent, fractionated into neutral and polar fractions, and composition determined by gas chromatography–mass spectrometry. Thirty-four fatty acids were quantified and their concentrations and percentages within each lipid fraction were calculated. Overall, saturated fatty acids (SFA) were predominant, accounting for 71 to 80% of fatty acids in neutral and polar lipid factions. There were marked differences in fatty acid composition between the lipid fractions (*P* < 0.001). The branched chain fatty acid (BCFA) concentration (15 to 18 µg) was almost twice as much as polyunsaturated fatty acids (PUFA) concentration found in the polar lipid fraction (8 to 9 µg; *P* < 0.001). Sperm with different freezability phenotypes only had a few differences in 22:0, 18:1 cis 9, and 14:0 13-methyl fatty acids (*P* ≤ 0.011). These results are significant because they reveal key understandings of fatty acid composition of sperm membrane and lay a foundation for the manipulation of membrane integrity, fluidity, and stability to advance the assisted reproductive technologies.

## Introduction

Artificial insemination (AI) has been commercially available for well over half a century, and during that time, beef and dairy producers have profited from the benefits of this technique. AI allows for the use of bulls that may be physically incapacitated or unable to perform natural breeding service, reduces labor costs by eliminating the need for maintaining bulls on the property for natural service, promotes the distribution and shipment of valuable genetics, and reduces the instances of disease transmissions by removing direct animal to animal contact. With these benefits, it is no surprise that production of bull semen constitutes a major agricultural business in the US and abroad. In 2018, nearly 22 million units of frozen dairy bull semen were sold domestically by American AI companies^1^. Costs such as genetic value premiums, semen costs, and bull maintenance are some of the top determinants of expected economic differences in natural service breeding systems verses AI breeding systems in regards to herd sizes and cow-to-bull ratios^2^. Nevertheless, a single bull can produce copious amounts of apparently normal sperm with good motility, but post-thaw viability of the sperm and cow pregnancy rates differ significantly.

While AI in equines is mostly dependent on fresh semen from the stallion, producers in the cattle industry utilize cryopreserved semen on a more regular basis. Cryopreservation is the process of cooling, and then, freezing sperm with the intention of preserving the integrity and function of the cell during the freeze–thaw process so it can be used in the future for AI. However, cryopreservation results in some significant damage of the sperm membrane, leading to cell death or the inability to function and fertilize^3–5^. In general, about 50% of the sperm will die during the freezing and thawing procedures^3^. Unfortunately, there’s no simple solution for rectifying this limitation to cryopreservation as molecular, cellular, and physiological determinants of sperm freezability are not fully understood.

Nevertheless, a promising step into solving the shortcomings of cryopreservation is through research in fatty acids. Among cellular differences of sperm, fatty acids are of great interest due to their responsibility in providing energy^6^ and structure to cells. Docosahexaenoic acid (DHA), for instance, has been documented in human models for its positive correlations with sperm motility^6^. In humans, compositional characteristics of the plasma membrane, including fatty acid composition, have been documented to modulate fluidity and freezability of sperm cells^7^. In bovine models, the membrane layers surrounding the nucleus and cytoplasm, as well as the tail, all contain critical lipids that play important roles in sperm physiology and integrity in both human and livestock species^8^. Saturated fatty acids (SFA), monounsaturated fatty acids (MUFA), and polyunsaturated fatty acids (PUFA), in addition to cholesterol and proteins, are vital components of sperm membrane and are important for successful fertilization^9^. In rabbit and bovine studies, greater proportions of PUFA and membrane proteins may lead to greater fluidity and sensitivity, resulting in sperm membrane damages^10^. Moreover, sperm fatty acid composition has been previously explored and was found to be an influencing factor of oligozoospermia and asthenozoospermia in human males^11^. This area of research is lacking, however, in bovines, and thus, the objective of the current study was to determine the fatty acid composition of sperm from Holstein bulls with good and poor freezability. We hypothesized that freezability is varied by the fatty acid composition in the sperm plasma membrane.

## Materials and methods

### Sperm freezability determination and experimental design

The sperm samples used in the current study were obtained from Alta Genetics, Inc. and the experiments did not involve live animals.

Sperm freezability was determined according to methods that have been previously described by other researchers^12^. Briefly, bulls (Alta Genetics, Watertown, WI, USA) were selected based on post-thaw viability data that were collected from 2008 to 2016. The semen quality database utilized 860 Holstein bulls and each bull was collected a minimum 15 times over a two-month period. Post-thaw viability of each bull was computed as an average of all bull samples from which the standard deviation was determined. The average post-thaw viability was 54.7% with a range of 33.02 to 67.2%. Bulls were arbitrarily placed into Good and Poor freezability phenotypes based on the average post-thaw viability percentage. The post-thaw viability and freezability categorization of bulls used in the current study is displayed in Table 1.

**Table 1:** Bull phenotype information.

Bull code	Freezability	Number of collections	Post-thaw viability (%)		
			Mean	Standard deviation	Difference from the population mean
1	Good	79	48.9	10.0	− 5.8
2		107	49.2	6.0	− 5.5
3		194	52.7	8.9	− 2.0
4		264	54.8	9.1	− 0.1
5		71	54.9	10.4	0.2
6		229	55.0	8.2	0.3
7	Poor	15	58.4	11.5	3.7
8		113	61.9	8.0	7.3
9		153	62.3	8.1	7.7
10		205	64.3	8.8	9.6
11		50	64.4	11.3	9.7
12		116	66.2	5.2	11.5

Bulls were housed at the same facility and were managed identically. Semen was collected by artificial vagina and a subsample was placed into a separate tube (500 × 10^6^ sperm per bull) and mixed with protease inhibitors. The subsample sperm were separated from the seminal plasma by centrifugation at 800×*g* for 15 min. Sperm were washed twice with cold PBS (GE Life Sciences, Logan, Utah, USA) and subsequently centrifuged at 700×*g* and 4 °C for 15 min after each wash. Following the second wash, the samples were aliquoted into 100 million sperm per tube and were stored in liquid nitrogen. The samples were shipped to Mississippi State University in a liquid nitrogen tank for fatty acid analysis.

### Lipid extraction

The cellular fatty acids were extracted using a previously described method^13^ with modifications. Frozen aliquots (10^8^ sperm/tube) were placed into their own receptible, then, diluted to 10^7^ cells. An aliquot from each bull containing 10^7^ sperm cells was pipetted into a reaction vial with 30 μL of 1.5 mg/mL methyl tridecanoate and 30 μL of 1.5 mg/mL tridecanoic acid as internal standards for neutral lipid (NL) and polar lipid (PL) fractions, respectively. Fatty acids were extracted in cold 500 μL of methanol for 10 min and subsequently in 250 µL of cold chloroform for 2 h. After 15,000×*g* centrifugation for 10 min at room temperature, the supernatant was transferred to a new 2-mL polypropylene microcentrifuge tube and the pellet was extracted again with an addition of 400 µL of water. This supernatant was combined with the previous one. The extract was allowed to separate overnight in a − 20 °C freezer. The bottom layer containing fatty acids was placed in an amber GC vial (Agilent Technologies, USA) and dried under a gentle nitrogen stream at 40 °C. The lipid residue was stored at − 20 °C.

### Lipid fractionation and fatty acid derivatization

Neutral and polar lipid fractions were separated as previously described^14^. Silica gel cartridges (100 mg/1 mL; Agilent Technologies, USA) were conditioned with 500 µL of methanol and 500 µL of chloroform in that order. Vacuum was used to obtain a 0.05 mL/min flow rate. Lipid residue was reconstituted with 200 µL of chloroform. Lipid solution in chloroform was pipetted into the cartridge and collected into a new amber GC vial. The lipids were eluted first with 200 µL of chloroform to obtain neutral lipids in a new vial. The vials were rinsed with 4 × 200 µL of chloroform and each rinse was used to wash the cartridge. A final wash with 300 µL of chloroform was also performed and combined in the same neutral lipid vial. The polar lipid fraction was eluted with 6 × 200 µL and a final 300-µL wash with methanol. Both lipid fractions were dried under a gentle nitrogen stream at 40 °C and stored in − 80 °C freezer. Dried lipid fractions were derivatized using a previously described method^15^ with modifications^16^. Lipids were saponified with aqueous 10-N KOH and methanol at 55 °C for 1.5 h. Liberated fatty acids were trans-esterified with 24-N H_2_SO_4_ and methanol for 1.5 h at 55 °C for 1.5 h. Fatty acid methyl esters (FAME) were extracted in hexane and stored at − 20 °C for GC–MS determination.

### Gas chromatography–mass spectrometry analysis

The fatty acid composition was determined by using an Agilent 7890A GC system equipped with a HP-88 capillary column (30 m × 0.25 mm × 0.20 µm), an autosampler, and a split/splitless injector (Agilent Technologies Inc., Santa Clara, CA, USA). The FAME were separated in a 20-min temperature-gradient program with helium as the carrier gas and quantified by an Agilent 5975C inert XL MSD with triple-axis mass detector in a selected ion monitoring mode. Fatty acid methyl esters were identified by comparing their retention times and mass spectra with authentic FAME standards and quantified by an internal standard calibration method. The gravimetric concentration of each fatty acid was calculated according to a previous study with correction for the difference in the molecular weight between FAME and their corresponding FA^17^. The normalized percentage of each FA was calculated by dividing gravimetric concentration by the total fatty acid concentration and multiplying with 100. The saturation index (SI) was calculated as a ratio of total SFA to total UFA (including MUFA and PUFA) concentration^17^.

### Statistical analysis

A generalized linear mixed model was used to analyze the data with freezability phenotype, lipid fraction, and their interaction as fixed effects. Analysis of variances was performed by the GLIMMIX procedure of SAS 9.4 (SAS Institute Inc, Cary, NC). Means, if different, were separated by the LSMEANS statement of the GLIMMIX procedure. Degree of freedom was estimated by the Kenward-Roger approximation. Actual probability values to determine statistical significance were reported. *P*-values < 0.05 were considered significant.

## Results

### Compositional characteristics of neutral and polar lipid fractions of sperms

Total of thirty-four fatty acids were detected and quantified for sperm across the two phenotypes and two lipid fractions using GC–MS. Total fatty acid concentrations were 268.45 and 238.44 µg per 10^7^ cells for Good and Poor phenotypes, respectively. Fatty acid concentrations of the NL and PL fractions ranged from 55.69 to 59.26 µg and 179 to 212 µg per 10^7^ sperm, respectively. There were 7 fatty acids in the SFA category, 15 fatty acids in the MUFA category, 8 fatty acids in the PUFA category, and 4 fatty acids in the BCFA category (Tables 2, 3).

**Table 2:** Fatty acid concentration (µg/10^7^ cells) in neutral (NL) and polar (PL) lipid fractions of 10^7^ sperms from bulls classified by Good and Poor freezability phenotypes.

Fatty acid	Good		Poor		SE	*P*_*p*_	*P*_*f*_	*P*_*x*_
	NL	PL	NL	PL				
**Saturated fatty acids (SFA)**	40.11	168.45	42.48	143.36	10.66	0.299	< 0.001	0.212
14:0	2.70	5.26	2.65	4.81	0.22	0.267	< 0.001	0.374
15:0	0.45	1.90	0.45	1.70	0.08	0.255	< 0.001	0.225
16:0	13.73	66.89	15.17	57.32	4.10	0.334	< 0.001	0.194
17:0	2.32	4.16	2.70	3.64	0.33	0.840	< 0.001	0.187
18:0	19.62	88.08	20.20	73.97	6.06	0.277	< 0.001	0.239
20:0	1.30	1.91	1.31	1.76	0.05	0.184	< 0.001	0.139
22:0	0.00	0.25^a^	0.00	0.17^b^	0.02	0.060	< 0.001	0.060
**Monounsaturated fatty acids (MUFA)**	13.24	16.48	14.30	12.09	1.93	0.399	0.791	0.174
14:1 cis 9	2.16	0.37	2.55	0.46	0.17	0.180	< 0.001	0.384
15:1 cis 9	1.03	0.80	1.07	0.47	0.27	0.591	0.135	0.490
16:1 cis 6	1.35	0.21	1.60	0.17	0.22	0.639	< 0.001	0.514
16:1 cis 9	0.61	0.76	0.38	0.45	0.14	0.067	0.456	0.788
16:1 cis 7	0.74	0.00	0.95	0.00	0.13	0.435	< 0.001	0.435
17:1 cis 10	2.08	0.56	2.30	0.41	0.38	0.924	0.000	0.626
18:1 trans 11	1.16	1.39	0.59	1.36	0.37	0.440	0.196	0.474
18:1 cis 9	1.90	10.09^a^	2.84	7.43^b^	0.90	0.352	< 0.001	0.058
18:1 cis 11	0.09	0.66	0.00	0.53	0.10	0.301	< 0.001	0.854
18:1 cis 12	0.19	0.18	0.21	0.11	0.09	0.768	0.501	0.628
18:1 cis un	0.47	0.07	0.65	0.11	0.30	0.714	0.130	0.807
19:1 cis 10	0.62	0.19	0.54	0.00	0.12	0.264	0.001	0.646
19:1 un	0.41	0.21	0.34	0.00	0.14	0.339	0.071	0.642
20:1 cis 11	0.22	0.00	0.21	0.00	0.05	0.853	0.000	0.853
22:1 cis 11	0.22	1.01	0.07	0.60	0.17	0.114	0.001	0.449
**Polyunsaturated fatty acids (PUFA)**	2.30	9.34	2.46	8.06	0.77	0.474	< 0.001	0.358
18:2 trans 9,12	0.29	0.65	0.79	0.32	0.27	0.763	0.84	0.139
18:2 cis 9,12	0.00	0.92	0.00	0.92	0.10	1.000	< 0.001	1.000
18:3 cis 6,9,12 (γ-linolenic acid)	0.31	0.00	0.46	0.00	0.07	0.316	< 0.001	0.316
20:2 cis 9,12	0.00	0.00	0.10	0.00	0.05	0.329	0.329	0.329
20:3 cis 8,11,14	0.49	0.14	0.52	0.00	0.13	0.715	0.004	0.526
20:4 cis 5,8,11,14	0.32	0.59	0.16	0.53	0.11	0.326	0.008	0.623
22:4 cis 7,10,13,16	0.58	1.83	0.15	2.04	0.26	0.685	< 0.001	0.235
22:6 cis 4,7,10,13,16,19	0.31	5.22	0.28	4.25	0.42	0.249	< 0.001	0.276
**Branch-chained fatty acids (BCFA)**	0.05	18.49	0.03	15.68	1.00	0.173	< 0.001	0.180
14:0 13-methyl	0.00	0.10^a^	0.00	0.04^b^	0.01	0.047	< 0.001	0.047
15:0 14-methyl	0.05	0.34	0.03	0.28	0.02	0.075	< 0.001	0.444
16:0 15-methyl	0.00	6.22	0.00	5.43	0.33	0.248	< 0.001	0.248
16:0 14-methyl	0.00	11.83	0.00	9.92	0.65	0.156	< 0.001	0.156
**Total fatty acids**	55.69	212.76	59.26	179.18	12.88	0.258	< 0.001	0.165
**Saturation index (SI)**	2.83	6.62	2.65	7.20	0.43	0.642	< 0.001	0.389

*P*_*p*_*, P*_*f*_*,* and *P*_*x*_: level of significance for freezability phenotype, lipid fraction, and their interaction.

*SE* pooled standard error, *un* represents an unknown isomer.

Saturation index = SFA/(MUFA + PUFA).

^a,b^If denoted by superscripts, means without common letters differ.

**Table 3:** Fatty acid percentage in neutral (NL) and polar (PL) lipid fractions of 10^7^ sperms from bulls classified by Good and Poor freezability phenotypes.

Fatty acid	Good		Poor		SE	*P*_*p*_	*P*_*f*_	*P*_*x*_
	NL	PL	NL	PL				
**Saturated fatty acids (SFA)**	72.49	78.96	71.38	79.99	1.85	0.983	0.001	0.568
14:0	4.93	2.56	4.48	2.68	0.28	0.572	< 0.001	0.320
15:0	0.80	0.91	0.76	0.95	0.04	0.965	0.001	0.322
16:0	24.75	31.41	25.49	31.99	0.60	0.287	< 0.001	0.898
17:0	4.14	1.97	4.51	2.04	0.30	0.485	< 0.001	0.619
18:0	35.51	41.06	33.91	41.26	1.26	0.584	< 0.001	0.481
20:0	2.36	0.93	2.23	0.98	0.08	0.634	< 0.001	0.269
22:0	0.00	0.12^a^	0.00	0.09^b^	0.01	0.065	< 0.001	0.065
**Monounsaturated fatty acids (MUFA)**	23.42	7.64	24.18	6.76	1.83	0.974	< 0.001	0.659
14:1 cis 9	3.84	0.20	4.39	0.26	0.32	0.341	< 0.001	0.441
15:1 cis 9	1.81	0.33	1.80	0.26	0.29	0.881	< 0.001	0.927
16:1 cis 6	2.39	0.11	2.67	0.09	0.29	0.667	< 0.001	0.619
16:1 cis 9	1.07	0.36	0.62	0.25	0.18	0.127	0.007	0.349
16:1 cis 7	1.30	0.00	1.62	0.00	0.20	0.449	< 0.001	0.449
17:1 cis 10	3.71	0.26	3.83	0.23	0.51	0.927	< 0.001	0.884
18:1 trans 11	1.98	0.65	0.99	0.76	0.52	0.411	0.155	0.306
18:1 cis 9	3.37^a^	4.67	4.78^b^	4.15	0.49	0.371	0.506	0.064
18:1 cis 11	0.16	0.35	0.00	0.30	0.09	0.276	0.019	0.552
18:1 cis 12	0.32	0.10	0.37	0.06	0.12	0.973	0.040	0.715
18:1 cis un	0.86	0.04	1.26	0.06	0.56	0.712	0.086	0.738
19:1 cis 10	1.11	0.06	0.89	0.00	0.12	0.239	< 0.001	0.501
19:1 un	0.73	0.07	0.52	0.00	0.16	0.401	0.001	0.661
20:1 cis 11	0.39	0.00	0.34	0.00	0.07	0.739	< 0.001	0.739
22:1 cis 11	0.38	0.45	0.11	0.34	0.10	0.075	0.157	0.447
**Polyunsaturated fatty acids (PUFA)**	4.00	4.66	4.40	4.50	1.09	0.914	0.729	0.801
18:2 trans 9,12	0.53	0.30	1.45	0.18	0.46	0.496	0.119	0.273
18:2 cis 9,12	0.00	0.48	0.00	0.51	0.05	0.790	< 0.001	0.790
18:3 gamma cis 6,9,12	0.53	0.00	0.80	0.00	0.14	0.339	< 0.001	0.339
20:2 cis 9,12	0.00	0.00	0.15	0.00	0.07	0.329	0.329	0.329
20:3 cis 8,11,14	0.83	0.08	0.94	0.00	0.21	0.940	0.001	0.665
20:4 cis 5,8,11,14	0.60	0.32	0.28	0.30	0.14	0.238	0.346	0.291
22:4 cis 7,10,13,16	1.02	0.86	0.30	1.15	0.29	0.463	0.246	0.093
22:6 cis 4,7,10,13,16,19	0.49	2.64	0.49	2.37	0.34	0.698	< 0.001	0.702
**Branch-chained fatty acids (BCFA)**	0.09	8.74	0.04	8.75	0.10	0.861	< 0.001	0.786
14:0 13-methyl	0.00	0.05^a^	0.00	0.03^b^	0.01	0.063	< 0.001	0.063
15:0 14-methyl	0.09	0.16	0.04	0.16	0.02	0.218	0.000	0.284
16:0 15-methyl	0.00	2.95	0.00	3.03	0.04	0.330	< 0.001	0.330
16:0 14-methyl	0.00	5.59	0.00	5.54	0.06	0.675	< 0.001	0.675

*P*_*p*_*, P*_*f*_*,* and *P*_*x*_: level of significance for freezability phenotype, lipid fraction, and their interaction.

*SE* pooled standard error, *un* represents an unknown isomer.

Saturation index = SFA/(MUFA + PUFA).

^a,b^If denoted by superscripts, means without common letters differ.

In the NL, SFA was the predominant FA category (*P* < 0.001), being almost 3 times greater than MUFA and 20 times greater than the PUFA (Table 2). The BCFA was the less abundant fatty acid (*P* < 0.001), ranging from 0.03 to 0.05 µg per 10^7^ cells. Within the SFA category, 18:0 was the most abundant fatty acid (19.62 to 20.2 µg/10^7^ sperm), followed by 16:0 (13.73 to 15.17 µg/10^7^ sperm). Of the MUFA category, 14:1 cis 9 was the most abundant fatty acid (2.16 to 2.55 µg/10^7^ sperm; *P* < 0.001), followed by 17:1 cis 10 (2.08 to 2.3 µg/10^7^ sperm) and 18:1 cis 9 (1.9 to 2.84 µg/10^7^ sperm). In the PUFA category, 18:2 trans 9,12 (0.29 to 0.79 µg/10^7^ sperm), 20:3 cis 8,11,14 (0.49 to 0.0.52 µg/10^7^ sperm), and 22:4 cis 7,10,13,16 (0.15 to 0.58 µg/10^7^ sperm) were the most abundant fatty acids (*P* < 0.001). The BCFA were not quantifiable in the NL, except for 15:0 14-methyl ranging from 0.03 to 0.05 µg per 10^7^ cells (Table 2).

As reported previously, the total fatty acid concentration in the PL fraction was much greater than that in the NL fraction (Table 2; *P* < 0.001). Although predominant fatty acids in the SFA category remained similar, the predominant patterns in other categories were different. Similar to the NL, SFA was the predominant category of FA in the PL, being almost 10 and 20 times greater than the MUFA and PUFA (*P* < 0.001), respectively. Also, 18:0 (74 to 88 µg) and 16:0 (57 to 67 µg) were the predominant fatty acids of the SFA category (*P* < 0.001). However, the BCFA concentration (15.68 to 18.49 µg) was almost twice as much as that of PUFA (8 to 9 µg; *P* < 0.001). In addition, in the MUFA category of the PL, 18:1 cis 9 was the predominant fatty acid (*P* < 0.001) with 7 to 10 µg, accounting for more than 50% of the MUFA concentration. Other major MUFA detected in the NL were not found in substantial concentration in the PL, even less than 18:1 trans 11 (1.4 µg; *P* < 0.001). In PUFA category of the PL, the 22:6 cis 4,7,10,13,16,19 (4.25 to 5.22 µg) and 22:4 cis 7,10,13,16 (1.8 to 2.0 µg) were the predominant fatty acids (*P* < 0.001). Additionally, these fatty acids were almost 16 and 3 times greater in the PL than in the NL, respectively (*P* < 0.001). However, 18:3 cis 6,9,12 (γ-linolenic acid) was not quantifiable in the PL fraction. Moreover, the BCFA was the second greatest category (15.7 to 18.5 µg; *P* ≤ 0.059) in the PL, much greater than that in the NL (*P* < 0.001).

Despite drastic differences in fatty acid concentrations between the NL and PL, the predominant patterns of fatty acid concentrations were translated into percentages (Table 3). Saturated fatty acids accounted for 71 to 80% of the total fatty acids across NL and PL. The PL had approximately 6 to 7% more SFA than the NL (*P* = 0.004). Within the SFA category, 16:0 accounted for 24 to 25% of total NL fatty acids and 31 to 32% of PL fatty acids. The 18:0 accounted for 34 to 35% of the NL fatty acids and 41% of the PL fatty acids. In the MUFA category, the 18:1 cis 9 accounted for 3 to 5% of fatty acids in the NL and PL; whereas 14:1 cis 9 and 17:1 cis 10 accounted for 3 to 4% of fatty acids in the NL, but only 0.2% in the PL (*P* < 0.001). Total MUFA contributed 23 to 24% of fatty acids to the NL but only 6 to 7% to the PL (*P* < 0.001). The PUFA contributed approximately 4 to 4.5% of fatty acids to both the NL and PL (*P* = 0.555). The 18:2 (trans 9,12), 18:3 (cis 6,9,12), 20:4 (cis 5,8,11,14), and 22:4 cis 7,10,13,16 had greater percentages in the NL than the PL (*P* < 0.001). However, 22:6 cis 4,7,10,13,16,19 had the greatest percentage in the PL (*P* < 0.001), contributing 2.4 to 2.6% of fatty acids to the PL. The BCFA accounts for approximately 8.7% to PL fatty acids with 16:0 14-methyl being greatest at 5.5% (*P* < 0.001); whereas, BCFA was only found at much lower percentage in the NL (0.04 to 0.09%; *P* < 0.001).

### Compositional differences in fatty acids of sperms with good and poor cryo-tolerance

There were 2-way freezability × fraction interactions for 22:0 (*P* = 0.060), 18:1 cis9 (*P* = 0.058), and 14:0 13-methyl (*P* = 0.047). For 22:0, there was no freezability difference in the NL (*P* = 1.000) between Good and Poor freezability groups (not quantifiable); whereas in the PL, the Good freezability had 0.08 µg or 0.03% more than the Poor freezability (*P* = 0.011). The 18:1 cis9 concentration did not differ in the NL (*P* = 0.464) but was 2.66 µg greater for the Good freezability in the PL (*P* = 0.049). However, the difference in concentration was not translated into a difference in percentage in the PL (*P* = 0.468). For 14:0 13-methyl, there was no freezability difference in the NL (*P* = 1.000) because this fatty acid was not quantifiable; however, in the PL, the Good freezability had 0.06 or 0.02% more than the Poor freezability (*P* = 0.007). There was an overall freezability effect on 16:1 cis9, with Good sperm having 0.54 µg/10^7^ more than Poor sperm (*P* = 0.067). Most other major fatty acids in SFA (16:0 and 18:0), MUFA (14:1 and 17:1), and PUFA (18:2 trans 9,12, 18:3 cis 6,9,12, 20:4 cis 5,8,11,14, and 22:4 cis 7,10,13,16) were not different between the Good and Poor freezability (*P* ≥ 0.075).

## Discussion

Cryopreservation of sperm is important for preservation of the male gametes of farm animals. It has assisted in improving the efficiency of reproduction of livestock through artificial insemination, thus, strengthening food security on a global scale. Sperm cryopreservation is also important for reproduction of other mammals including humans and endangered species. Furthermore, cryopreservation is essential in biomedicine through the preservation of germ cells for cancer patients before they undergo chemotherapy. Nevertheless, drawbacks to cryopreservation are evident as efficiency of sperm freezability is low and the sperm that endure the process still suffer significant structural damage that hampers post-thaw viability. These hinderances associated with cryopreservation are further compounded by the fact that there exists significant gaps in the knowledge and technology bases for efficient cryopreservation of sperm, and thus, the objective of this study was to ascertain the lipidome of bulls with established sperm freezability phenotypes.

The number of the FA and abundance of the fatty acid categories in the current study differ from those in various studies reported in the literature. One study successfully identified 26 fatty acids. Studies utilizing human spermatozoa also vary in success with a total of nine, twenty-nine, and ten FA when utilizing gas chromatography methods^11,18–20^. In animal studies, a total of 18 and 27 FA were also detected in bull and stallion sperm respectively^21,22^. In the current study, we identified a total of 34 FA in the categories of SFA, MUFA, PUFA, and BCFA. Several FA were found in this study, including 18:1 cis 11, 18:2 trans 9,12, and 22:4 cis 7,10,13,16, that were not identified in human spermatizoa^18,19^. The differences in FA identification may be a result of different separation and detection techniques employed in these studies and this needs to be addressed in further studies. Mass spectroscopy was used to detect fragmented ions in the current study, whereas, flame ionization detection was used in most cited studies. Mass spectrometry offers detailed spectral information on the potential structure of fatty acids even though some may be eluted at the same time (same retention times). The flame ionization detection, on the other hand, only allows for compound identification by retention time; therefore, co-eluted fatty acids cannot be determined separately^23^.

Moreover, most studies on FA composition of sperm used GC system with a flame ionization detection only reporting peak area, which was less accurate than ion abundance^23^. Most authors calculated FA percentages (g per 100 g of total fatty acids or lipids) by using peak area, instead of building actual standard curves for the determination of gravimetric concentrations^17^. As a result, most authors reported FA percentages, instead of concentrations. Of a few studies reporting FA concentrations in sperm, one reported concentrations of 9, 5, 3, and 8 nmol/10^7^ for 16:0, 18:0, 18:1 cis9, and 22:6 FA in human sperm, which were approximately 2.3, 1.5, 0.9, 2.6 µg/10^7^ sperm^24^. These concentrations were much lower than our findings even though these authors did not separate sperm lipids into neutral and polar fractions. Another study reported that phospholipids in human sperm were at 10 to 20 nmol/10^7^ sperm, which was 10 times greater than what was reported by previous publications^24,25^. The differences perhaps can be explained by the flame ionization detection analytical techniques employed by the previous study with mass spectroscopy in the current study^24^. Moreover, the previous study used two volumes of chloroform in one volume of methanol to extract sperm lipids, different from chloroform/methanol (1:2 v/v) used in the current study^24^. Sperm lipids are located primarily in the cell membrane in the polar lipid fraction, as reported in the current study. Using less methanol would prevent efficient recovery of fatty acids in the polar lipid fraction, e.g. membrane lipids.

Previous reports showed that SFA is the most abundant fatty acids in human sperm with percentages ranging from 42 to 49%^11,18,26^. Polyunsaturated fatty acids were the second predominant FA category in these reports, ranging from 30 to 36%. Monounsaturated fatty acids were the least abundant FA category in human sperm, ranging from 14 to 23%. However, these results were different from the current study and other findings^21^. The SFA are still the most abundant FA in bull sperm; however, the proportion of MUFA overtakes that of PUFA to be the second predominant category in bull sperm. These differences can be potentially explained by species. For example, bulls are ruminants having the ability to biohydrogenate dietary PUFA to SFA, which are subsequently desaturated by ∆^9^-desaturases to MUFA^27^, while humans and other monogastric species do not have this capability. Previous findings reported that the percentages of SFA, MUFA and PUFA were 45%, 36%, and 19%, respectively^21^. Compared to the current study, the SFA percentage reported was 31% less than that of a different study also performed using Holstein spermatazoa^21^. The MUFA category is comparable between what was reported by and in the current study^21^. However, only 4 to 5% was reported to be PUFA in the current study, 14% less than what these authors reported. However, approximately 9% branch-chained fatty acids (BCFA) was also reported in the current study. The BCFA, although having similar ion fragment patterns to SFA, are eluted in the regions of MUFA and PUFA. The detection by FID usually mistakes these BCFA as PUFA. The roles of BCFA in sperm freezability and fertility are unknown. Nevertheless, because of the bulkiness of their structures, they are thought to behave similarly to PUFA in their packing into cellular membrane^28^. The most abundant FA in studies cited above was 16:0, ranging from 23 to 30%, similar to proportion of 16:0 reported in the current study. However, 18:0 was the most abundant FA in the current study (34 to 41%).

Of a few studies separating neutral and polar lipids, it was reported that SFA was predominant in the NL fraction with 54%, which is similar to what was reported in the current study^22^. However, there is a difference in PL fraction between the current study and others. The current study, so far, is the first to report that bull sperm polar lipid fraction is highly saturated, with approximately 79 to 80% SFA, in contract to a stallion study reporting only 30% of SFA in the PL fraction^22^. These authors also reported 64% of PUFA in the PL fraction, whereas there was only 4 to 5% of PUFA in current study. Although being similar to a different study, where it was reported that 59% of PUFA in the phospholipids of ejaculated sperm, 55% was found to be 22:6^29^. However, many FA in the SFA and MUFA categories were not detected in the stallion study, some of which are eluted in the PUFA regions of the GC chromatogram, which may not be able to be detected with the FID^22^. In addition to potential species differences, the extraction protocol used for the stallion study utilized chloroform/methanol (2:1 v/v) for FA extraction, instead of chloroform/methanol (1:2 v/v) used in the current study. This potentially missed some polar lipids, as discussed previously. Moreover, instead of using silica gel, these authors used aminopropyl cartridges, which is slightly less polar and more selective. Silicic acid (silica gel; though density and particle size are unknown) is similar to what was used in the current study^29^. However, these authors employed 2-D thin-layer chromatography for fractionation with hexane-diethyl ether-acetic acid (20:80:1 v/v/v) and chloroform/methanol (1:9 v/v) for eluting lipid classes. This method is more selective than chloroform/methanol alone and was specified by the authors for the purposes of separating phosphorus-containing lipids. The current study employed two internal standards, one for NL and one for PL fraction. Exhausted recovery was checked by internal standard total ion abundance and the residual FA in the last rinse. There was no crossover of the two lipid fractions.

In future studies, nuclear magnetic resonance spectroscopy (NMR) could be utilized to determine the differences that are present in low and high freezability sperm samples because it is capable of detecting a variety of metabolites, to include fatty acids^30,31^. In an early study on human sperm and seminal plasma extracts, it was found that NMR could detect high quantities of double bonds within the sample whereas the matrix-assisted laser desorption and ionization time-of-flight mass spectrometry (MALDI-TOF MS) was capable of detecting peaks within the sample^32^. In early studies such as this one, samples had to be relatively pure in order to have successful elucidation^33^. Researches were then able to determine what fatty acid was detected at each peak. In this case, the NMR was not capable of showing differences in the samples after cryopreservation had taken place. In a more recent study, the metabolic profile of fresh turkey sperm was evaluated over the course of three different reproductive ages; 32, 44, and 56 weeks^30^. It was found that the diunsaturated fatty acids increased in level from the 32 to 56 weeks but the PUFA level decreased at 56 weeks of age. The decrease in PUFA is associated with decreased sperm quality, membrane destabilization, and peroxidation of membrane lipids^30,34^. Technologies such as NMR can aid in the exploration and identification of sample contents but the complexity of the sample and desired sensitivity of the technique need to be considered.

Fatty acids play a vital role in the lipid membranes of cells and are essential for signal transduction, metabolism and growth^35^, membrane fluidity^7,36^, and regulation of gene expression^37^. Therefore, lipid composition of bull sperm can be used to estimate freezability phenotype of the spermatozoa^38^. As SFA are the most abundant FA group in sperm, composition of this group plays an important role to determine freezability phenotype of the sperm. Previous studies showed that concentrations of 16:0 and 18:0 were increased after the freezing–thawing process in human sperm^11,39^. Furthermore, SFA were higher in asthenozoospermic and oligozoospermic patients than in normozoospermic men. In our study, there was no difference in SFA between good and poor freezability sperm, except that arachidic acid (22:0) was greater in the PL fraction of the Good freezability sperm. Saturated fatty acids, especially the long-chained SFA such as 22:0, modify the fluidity of cellular membrane by increasing the energy (higher melting point) required for maintaining fluidity. In addition, it has been reported that MUFA was inversely related to post-thaw sperm viability in human sperm and these reports suggested that oleic acid may be a substrate of lipid oxidation^19,40^, which might cause a decrease in sperm motility^24,26^. However, in the current study, 18:1 cis9 (oleic acid) was more abundant in the PL fraction of Good freezability sperm. In contrast to what was suggested by reports, supplementation of oleic acid reduced ROS production and increased ATP generation for hyperactivation of bull sperm^41^. Although greater PUFA percentage creates more membrane fluidity and flexibility^42,43^, allowing for more resistance to cryodamage^19^, PUFA is the leading cause of lipid peroxidation due to the large number of double bounds^7^. Nevertheless, no difference in PUFA was reported in the current study.

## Conclusions

Although there were differences in extraction, fractionation, and determination methods between the current study and others in the literature, the current study is the first to report that bull sperm fatty acid composition is highly saturated with the majority of FA being located in the PL fraction, and so, more likely on the cellular membrane of the sperm. Branch-chained fatty acids were also quantified in a greater percentage than PUFA, raising interesting questions about the roles of BCFA in packing into sperm membrane and modulate membrane fluidity. Arachidic and oleic acids were greater in the PL fraction of the Good sperm, which is most likely related to fluidity modulation and protection against oxidation during freezing and after thawing. These findings differ from those reported for other species including humans. In the end, the current study sheds light onto the roles of these fatty acids in membrane fluidity, cellular oxidative stress, and potential impacts on post-thaw viability of sperm.
